# Using Sex‐specific Cutoffs for High‐sensitivity Cardiac Troponin T to Diagnose Acute Myocardial Infarction

**DOI:** 10.1111/acem.14098

**Published:** 2020-08-26

**Authors:** W. Frank Peacock, Brigitte M. Baumann, E. Joy Rivers, Thomas E. Davis, Beverly Handy, Christopher W. Jones, Judd E. Hollander, Alexander T. Limkakeng, Abhi Mehrotra, Martin Than, Louise Cullen, André Ziegler, Carina Dinkel‐Keuthage

**Affiliations:** ^1^ From the Department of Emergency Medicine Baylor College of Medicine Houston TX USA; ^2^ the Department of Emergency Medicine Cooper Medical School of Rowan University Camden NJ USA; ^3^ Agent representing Roche Diagnostics Indianapolis IN USA; ^4^ the Indiana University School of Medicine Indianapolis IN USA; ^5^ the Department of Laboratory Medicine University of Texas MD Anderson Cancer Center Houston TX USA; ^6^ the Department of Emergency Medicine Thomas Jefferson University Philadelphia PA USA; ^7^ the Division of Emergency Medicine Duke University Durham NC USA; ^8^ the Department of Emergency Medicine University of North Carolina School of Medicine Chapel Hill NC USA; ^9^ the Emergency Department Christchurch Hospital Christchurch New Zealand; ^10^ the Department of Emergency Medicine Royal Brisbane and Women's Hospital Brisbane QLD Australia; ^11^ Roche Diagnostics International Ltd Rotkreuz Switzerland; ^12^ and Roche Diagnostics GmbH Penzberg Germany

High‐sensitivity cardiac troponin (hs‐cTn) assays facilitate early decision making in acute myocardial infarction (AMI).^1^ The accuracy of these assays now allow sex‐specific differences in levels to be detected within healthy populations. It is thought that differences in plasma levels of cardiac troponin (cTn) are due to sex‐specific variations in body composition and cardiac physiology^2^ and that estrogen may also play a part.^3^ However, the clinical relevance of this remains unclear.^4^ Women presenting with suspected acute coronary syndrome (ACS) are less frequently diagnosed, have poorer outcomes,^5^ and are more likely to have atypical symptoms than men.^6^ The requirement for sex‐specific cutoffs may vary depending on the troponin assay, intended use, AMI type, and clinical performance estimate being assessed. Previously proposed sex‐specific hs‐cTnT assay cutoffs (females, 14 ng/L; males, 22 ng/L) did not alter sensitivity for AMI versus the overall cutoff (19 ng/L), but resulted in slightly lower specificity for AMI in females and higher specificity in males.^4^ However, it is uncertain whether sex‐specific cutoffs improve hs‐cTnT assay diagnostic performance.

We investigated the impact of sex‐specific cutoffs on performance of the Elecsys® Troponin T Gen 5 (Roche Diagnostics GmbH, Mannheim, Germany) hs‐cTnT assay for diagnosing AMI and predicting 30‐day major adverse cardiac events (MACE). Exploratory analyses were performed using data from two patient cohorts enrolled in prospective U.S. studies:^4^, ^7^ an ACS cohort of 1,679 patients (median age = 55 years; 48.4% females), enrolled at 15 U.S. EDs from 2011 to 2015,^4^ and a reference range study^7^ of 1,301 healthy volunteers.

For our analyses, inclusion criteria included age ≥ 21 years, emergency department presentation with ACS symptoms, and provision of a baseline blood sample within 24 hours of symptom onset. Exclusion criteria included AMI in the healthy cohort, transfer from another medical facility, surgery or hospitalization within prior 3 months, cardioversion or defibrillation within prior 3 months, cardiogenic shock, and self‐declared pregnancy in the ACS cohort. Following application of these criteria, our ACS cohort analyses included 1,600 patients with an initial hs‐cTnT result and 1,415 with a 3‐hour hs‐cTnT result. All patients were adjudicated by a clinical events committee using all available clinical information according to the third universal definition of AMI.^8^

To assess the impact of sex‐specific versus overall cutoffs on hs‐cTnT assay performance, we evaluated the sensitivity, specificity, negative predictive value (NPV), and positive predictive value (PPV) of AMI diagnosis. We also calculated rates of 30‐day MACE (any post‐discharge death, AMI, or urgent revascularization) in patients with a 3‐hour hs‐cTnT result above the overall and sex‐specific cutoffs by sex and AMI diagnosis. Conditional random forest modeling was used to assess which of the following factors were associated with elevated 3‐hour hs‐cTnT results: AMI diagnosis, age, sex, body mass index (BMI), systolic/diastolic blood pressure, race, ethnicity, current smoker, and other cardiovascular risk factors and that may predict AMI diagnosis—initial hs‐cTnT result, 3‐hour hs‐cTnT result, percent and absolute difference between initial and 3‐hour hs‐cTnT results, age, sex, BMI, current smoker, and other cardiovascular risk factors.

Analyses were performed using R (version 3.4.0, R Foundation for statistical computing, Vienna, Austria) and SAS (version 9.4, SAS Institute, Cary, NC) software.

**Figure 1:**
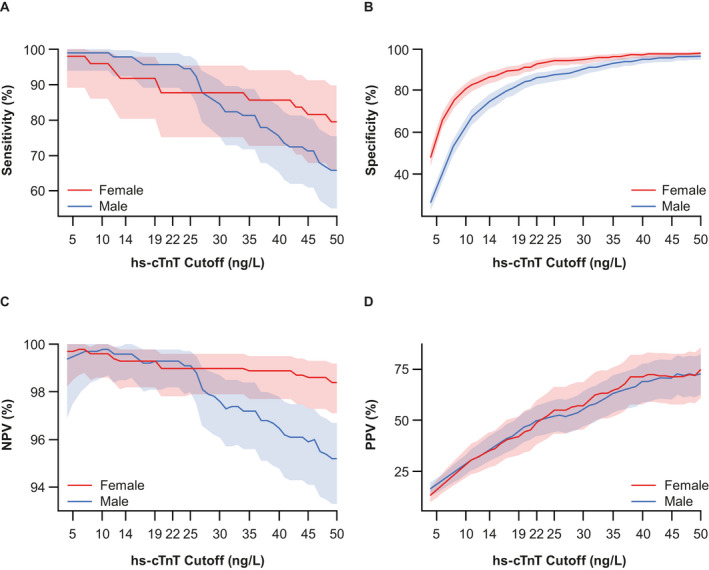
Diagnostic performance of the hs‐cTnT assay for the 3‐hour draw using sex‐specific 99th‐percentile URL cutoffs for (**A**) sensitivity, (**B**) specificity, (**C**) NPV, and (**D**) PPV. hs‐cTnT = high‐sensitivity cardiac troponin T; NPV = negative predictive value; PPV = positive predictive value; URL = upper reference limit. Overall cutoff 19 ng/L; sex‐specific cutoffs 14 ng/L (female) and 22 ng/L (male).

Our findings suggested that using sex‐specific versus overall cutoffs did not influence the sensitivity or NPV of the assay; however, specificity and PPV were decreased in females and increased in males (Figure 1). In females, sensitivity and specificity, respectively, were 91.8 and 86.9% with the 14 ng/L cutoff and 91.8 and 90.2% with the 19 ng/L cutoff. In males, sensitivity and specificity, respectively, were 95.6 and 86.3% with the 22 ng/L cutoff and 95.6 and 83.0% with the 19 ng/L cutoff. Two of 63 females diagnosed with AMI had an initial hs‐cTnT result between the female‐specific and overall cutoffs; thus, using the lower sex‐specific cutoff would increase the early/baseline AMI diagnosis rate by an absolute 3.2% in females. Three of 101 males diagnosed with AMI had an initial hs‐cTnT result between the overall and male‐specific cutoffs; thus, using the higher sex‐specific cutoff would decrease the early AMI diagnosis rate by an absolute 3.0% in males.

No patient diagnosed with AMI had a 3‐hour hs‐cTnT result between the respective sex‐specific and overall cutoffs; thus, sex‐specific cutoffs would have no impact on sensitivity by that time point. Among patients not diagnosed with AMI, 21 females and 21 males had a 3‐hour hs‐cTnT result between the respective sex‐specific and overall cutoffs, highlighting the potential impact of sex‐specific cutoffs on specificity. Three of these 21 females had a 30‐day adverse cardiac event (one death 19 days after enrollment and two non‐MI cardiac events on days 2 and 12 after enrollment), 15 had no further cardiac events, and three were lost to follow‐up. One of the 21 males had an adverse cardiac event, 19 had no further cardiac events, and one was lost to follow‐up.

Among patients with 3‐hour hs‐cTnT results above the overall and sex‐specific cutoffs, rates of 30‐day MACE for patients with AMI were 13% to 14% for females (all AMI diagnosis 7/49 [14%]; overall and sex‐specific cutoffs 6/45 [13%]) and 7% for males (all AMI diagnosis 6/91 [7%]; overall and sex‐specific cutoffs 6/87 [7%]). For patients without AMI, rates were 6% to 7% for females (overall cutoff 4/62 [6%]; sex‐specific cutoff 6/83 [7%]) and 1% for males (overall cutoff 1/109 [1%]; sex‐specific cutoff 1/88 [1%]).

Reviewing the association between patient characteristics by random forest modeling, AMI diagnosis, age, sex, and history of congestive heart failure were determined to have the highest importance scores for 3‐hour hs‐cTnT result (mean scores = 0.913, 0.117, 0.055, and 0.043, respectively). Initial and 3‐hour hs‐cTnT results were determined to have the highest importance scores for AMI diagnosis (0.134 and 0.114, respectively), followed by the percentage difference between initial and 3‐hour hs‐cTnT results (0.033).

Recent controversies surrounding sex‐specific cTn assay cutoffs and the underdiagnosis of AMI in females^9^ highlight the need for more data, a broader understanding of assay users’ requirements, and well‐defined acceptance criteria. Our analysis found the diagnostic performance of the hs‐cTnT assay was not substantially impacted when sex‐specific cutoffs were applied to serial results: sensitivity was not altered, and specificity was slightly lower in females but higher in males. We observed clinically relevant sex‐specific differences in hs‐cTnT results in healthy participants, suggesting a potential role in long‐term prognosis. Yet, we observed no age‐ or sex‐specific hs‐cTnT differences in patients diagnosed with AMI. However, the difference in distribution of results between patients with/without an AMI was greater in females versus males. It should be noted that patient management was based on AMI diagnosis using the U.S.‐specific hs‐cTnT overall 19 ng/L cutoff; a lower hs‐cTnT overall cutoff of 14 ng/L is used in the rest of the world. These findings suggest that sex‐specific cutoffs for hs‐cTnT are not required for AMI diagnosis in patients with suspected ACS.

Using sex‐specific cutoffs did not substantially impact hs‐cTnT assay diagnostic performance; however, among the 42 patients not diagnosed with AMI who had a 3‐hour hs‐cTnT result between the sex‐specific and overall cutoffs, 14% of females had a 30‐day MACE compared with 5% of males. Although based on a small number of events, and potentially influenced by selection bias, this finding is of some concern and its clinical significance should be explored.

The magnitude of our findings is less than those of the much‐cited study by Shah et al.,^10^ on which the call for sex‐specific cutoffs is principally based. In that study, 80 (16%) and 111 (22%) females were classified as having an AMI using overall and sex‐specific cutoffs for hs‐cTnI, respectively, compared with 55 (11%) females using a contemporary troponin I assay. In contrast, there was only a slight increase in the number of males classified as having an AMI using a hs‐cTnI (overall cutoff, *n* = 142 [23%]; sex‐specific cutoff, *n* = 131 [21%]) versus a contemporary cTnI assay (*n* = 117 [19%]) troponin I assay. These findings may be due to the greater difference between the sex‐specific (female, 16 ng/L; male, 34 ng/L) and overall (26 ng/L) cutoffs for hs‐cTnI, compared with hs‐cTnT.

Our analysis included data concurrently collected from a large U.S. reference cohort, enabling us to account for multiple confounding factors. Study limitations included the non‐standardized workup used for AMI diagnosis (although reflective of real‐world practice) and the low number of AMIs observed in females (*n* = 49 at the 3‐hour draw). Our results are specific to the Elecsys Troponin T gen 5 assay, and the analysis included only U.S. participants; these findings will need to be validated across different troponin assays and countries. It is unclear what impact clinician judgment or factors such as ECG interpretation would have on AMI diagnosis rates.

When using serial hs‐cTnT testing at admission and 3 hours later, sex‐specific cutoffs did not change the rate of AMI diagnosis at initial presentation. However, based on serial results at 3 hours, 30‐day MACE rates showed sex‐specific differences. Efforts to prevent underdiagnosis of AMI in females should ensure the use of good clinical judgment, serial hs‐cTnT measurements, and appropriate use of the 99th‐percentile upper reference limit, which is still not used in many U.S. centers.

We thank the individuals who participated in this study as well as the study investigators and their staff. Medical writing support was provided by David Evans, PhD, and Thomas Burton, BMBS (Gardiner‐Caldwell Communications, Macclesfield, UK) and was funded by Roche Diagnostics International Ltd, Rotkreuz, Switzerland. COBAS, COBAS E, and ELECSYS are trademarks of Roche.
